# Novel *KRIT1/CCM1* and *MGC4607/CCM2* Gene Variants in Chinese Families With Cerebral Cavernous Malformations

**DOI:** 10.3389/fneur.2018.01128

**Published:** 2018-12-21

**Authors:** Kang Wang, Dengchang Wu, Baorong Zhang, Guohua Zhao

**Affiliations:** ^1^Department of Neurology, The First Affiliated Hospital, Zhejiang University School of Medicine, Hangzhou, China; ^2^Department of Neurology, The Second Affiliated Hospital, Zhejiang University School of Medicine, Hangzhou, China; ^3^Department of Neurology, The Fourth Affiliated Hospital, Zhejiang University School of Medicine, Yiwu, China

**Keywords:** cerebral cavernous malformation, *CCM1*, *CCM2*, novel, genetic variant

## Abstract

Familial cerebral cavernous malformations (CCMs) are autosomal dominant disorders characterized by hemorrhagic strokes, recurrent headache, epilepsy, and focal neurological deficits. Genetic variants in *KRIT1/CCM1, MGC4607/CCM2*, and *PDCD10/CCM3* genes contribute to CCMs. The clinical information of two Chinese families with CCMs was collected. MRI and video-electroencephalography were performed. Genetic variants of *CCM1, CCM2*, and *CCM3* genes were investigated by exome sequencing. The patients were presented with recurrent epilepsy or headache. Susceptibility-weighted images of brains showed many dark dots, while video-electroencephalography revealed many spikes from multiple brain regions of patients. Exome sequencing revealed a novel *CCM1* genetic variant (c.1599_1601TGAdel, p.Asp533del) and a novel *CCM2* genetic variant (c.773delA, p.K258fsX34) in Family one and Family two, respectively; cosegregation existed in these two families. The two family members presented typical CCMs symptoms. These two novel genetic variants in *CCM1* and *CCM2* genes were the causation of CCM in the two Chinese families, and our data enriched the genetic variant spectrum of CCM genes.

## Introduction

Cerebral cavernous malformations (CCMs) are vascular anomalies characterized by densely packed tortuous microvessels outlined with deficient interstitial brain parenchyma. They are the second most prevalent type of vascular malformation, accounting for 10–15% of all vascular malformations in the central nervous system (CNS). These dysplastic capillaries are mainly present in the brain and spine cord, but they may also affect the skin and liver, with increased propensity to leak and rupture, causing hemorrhagic strokes, recurrent headache, epilepsy, and focal neurological deficits (1). CCMs can occur in a sporadic or autosomal dominant fashion (Familial cerebral cavernous malformations, FCCM), and the latter tends to have multiple lesions as a consequence of inherited loss-of-function genetic variants, predominantly in any of the *KRIT1/CCM1* (Krev interaction trapped 1), *MGC4607/CCM2* (cerebral cavernous malformation 2), or *PDCD10/CCM3* (programmed cell death protein 10) genes (2–4). The prevalence of genetic variants in the three genes is unknown, although there are several cases reported in different populations like Italian (5, 6), French (7), Spanish (8), Germany (9, 10), and Japanese (11). There are a few studies reporting *CCM1* genetic variants, but only one *CCM2* genetic variant was reported in Chinese patients (12–24).

In this study, we described the clinical and neuroradiological findings in two Chinese families with CCMs and identified two novel genetic variants in *CCM1* and *CCM2* genes, respectively.

## Material and Methods

### Subjects

Two pedigrees of the two Chinese families with CCMs were enrolled. This study was approved by the Institutional Review Board of Zhejiang University School of Medicine, and written informed consent was received from all subjects or their family members. The detailed medical history of the family members from the two pedigrees was obtained. Brain MRIs, including susceptibility-weighted images (SWI) and video-electroencephalography (VEEG), were performed in some cases.

### Genetic Analysis

Blood samples were collected from all members of these two families, and genomic DNA was extracted using commercial kits and then stored at −20°C. Exome sequencing was performed for *CCM1, CCM2*, and *CCM3* genes, which were sequenced in the two probands by Axeq Technologies (Precision Gene Technologies, Beijing, China). Briefly, Agilent SureSelect v6 reagents were used to capture exons, and Illumina HiSeq X Ten platform was used for sequencing as previously reported (25). Variants identified by sequencing were analyzed for cosegregation in the family members by Sanger sequencing, and polymorphism was excluded by 100 normal controls.

## Results

### Clinical Investigation

#### Family One

**Figure 1:**
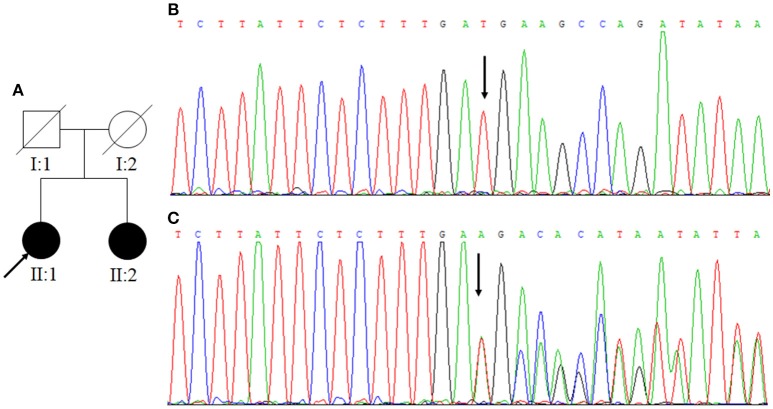
**(A)** Family One. Arrow indicates the proband; square represents a male, while circles represent females. The filled symbols mean CCM and slash indicates deceased. **(B)** The normal control sequences of *CCM1* gene. **(C)** The genetic variant of c.1599_1601TGAdel (p.533Aspdel). Affected nucleotide is indicated with an arrow.

**Figure 2:**
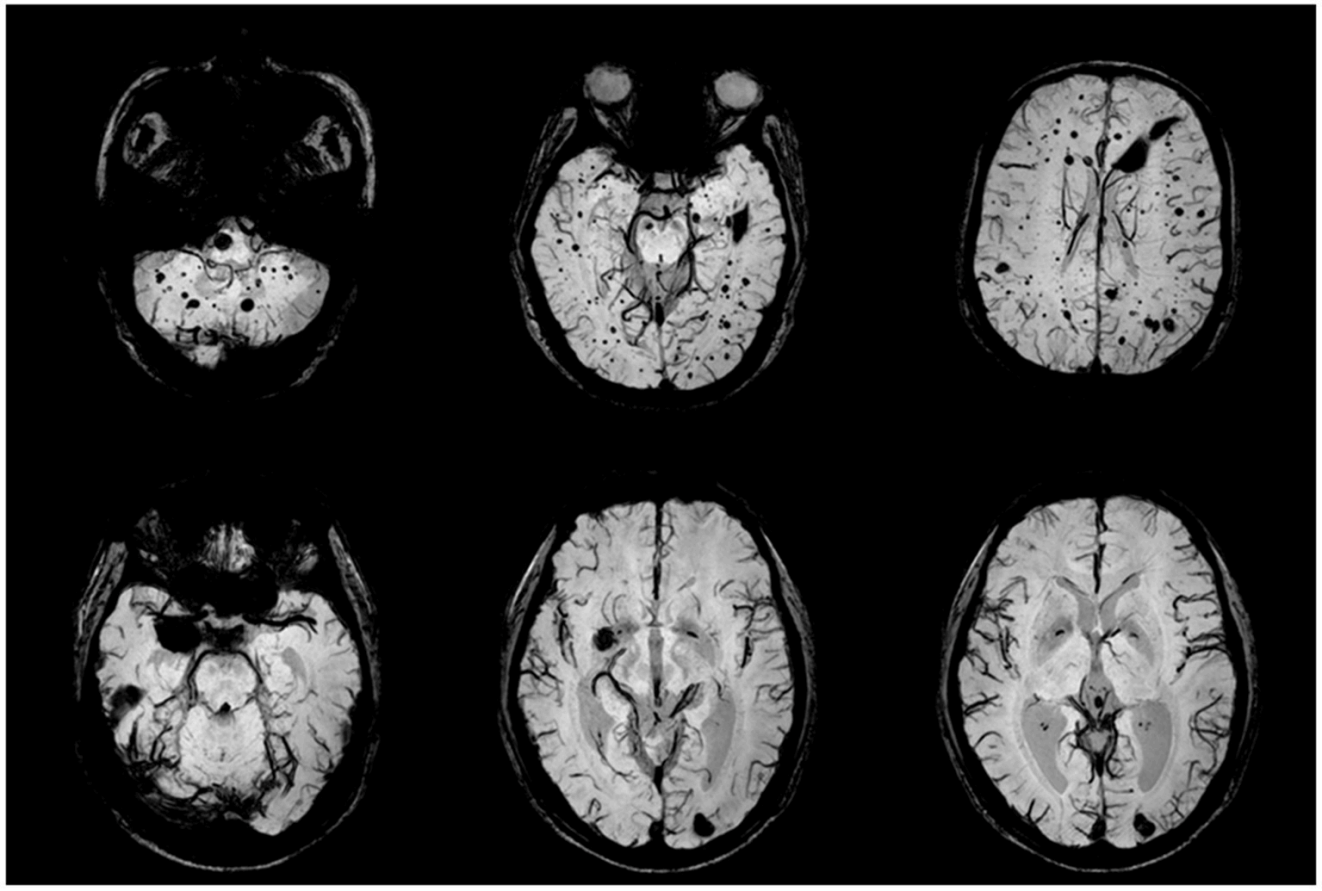
CCM lesions in Family One detected by SWI-MRI. The proband's SWIs (II:1) revealed dot and patchy low intensity lesions on temporal lobe, cerebellum and thalamus (upper row). SWI (II:2) showed 2 popcorn-like lesions in left occipital region and right temporal pole, which were very dark, suggesting chronic bleeding.

The proband II:1 (Figure 1A) was a 59-year-old woman who visited our clinic for recurrent episodes with consciousness loss followed by whole body stiffness and shaking movements for 3 years. These attacks occurred 5 to 8 times per year without any medication treatments because the seizures happened during the sleeps. No minor episodic events were reported. SWI of brain MRI taken on July 3, 2017 showed hundreds of dark dots and patches in different sizes distributed across the cerebral hemispheres, cerebellum, and brainstem, which indicates multiple cavernous malformations (Figure 2, upper row). VEEG showed abundant spikes that originated from multiple brain regions (data not shown). Her memory declined, and she suffered insomnia over the past 2 years. Habitual nasal bleeding was denied. On physical exams, no vascular malformation was found on the skin. Routine blood examinations were normal.

The proband's younger sister II:2 complained of chronic intensified and aggravated headaches for over 10 years. T2-weighted images of MRI showed 2 popcorn-like lesions in left occipital region and right temporal pole, and these lesions were hypo-intensities on SWI; those findings suggested chronic bleeding (Figure 2, lower row). No other illnesses or discomforts were reported. The proband's father (I:1) passed away due to a lethal heart attack at the age of 52 years, and her mother died of severe head trauma in a car accident at the age of 60 years. No cutaneous abnormalities, headache, stroke, or convulsion were recorded in the proband's parents.

#### Family Two

**Figure 3:**
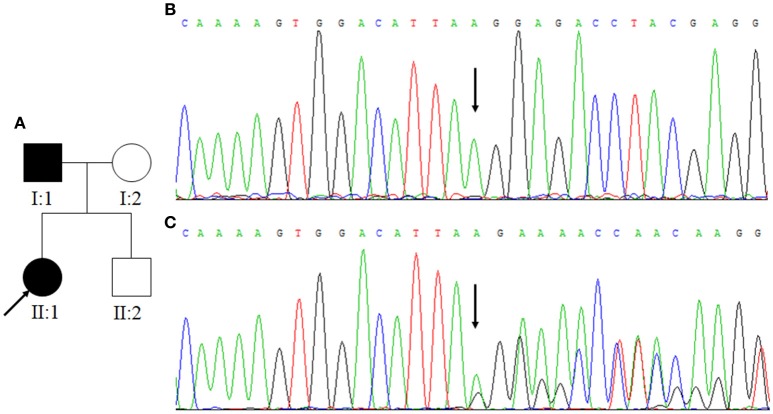
**(A)** Family Two. Arrow indicates the proband. Squares indicate males while circles indicate females. The filled symbols represent CCM. **(B)** The normal control sequences of *CCM2* gene. **(C)** The genetic variant of c.773delA. Affected nucleotide is indicated with an arrow.

**Figure 4:**
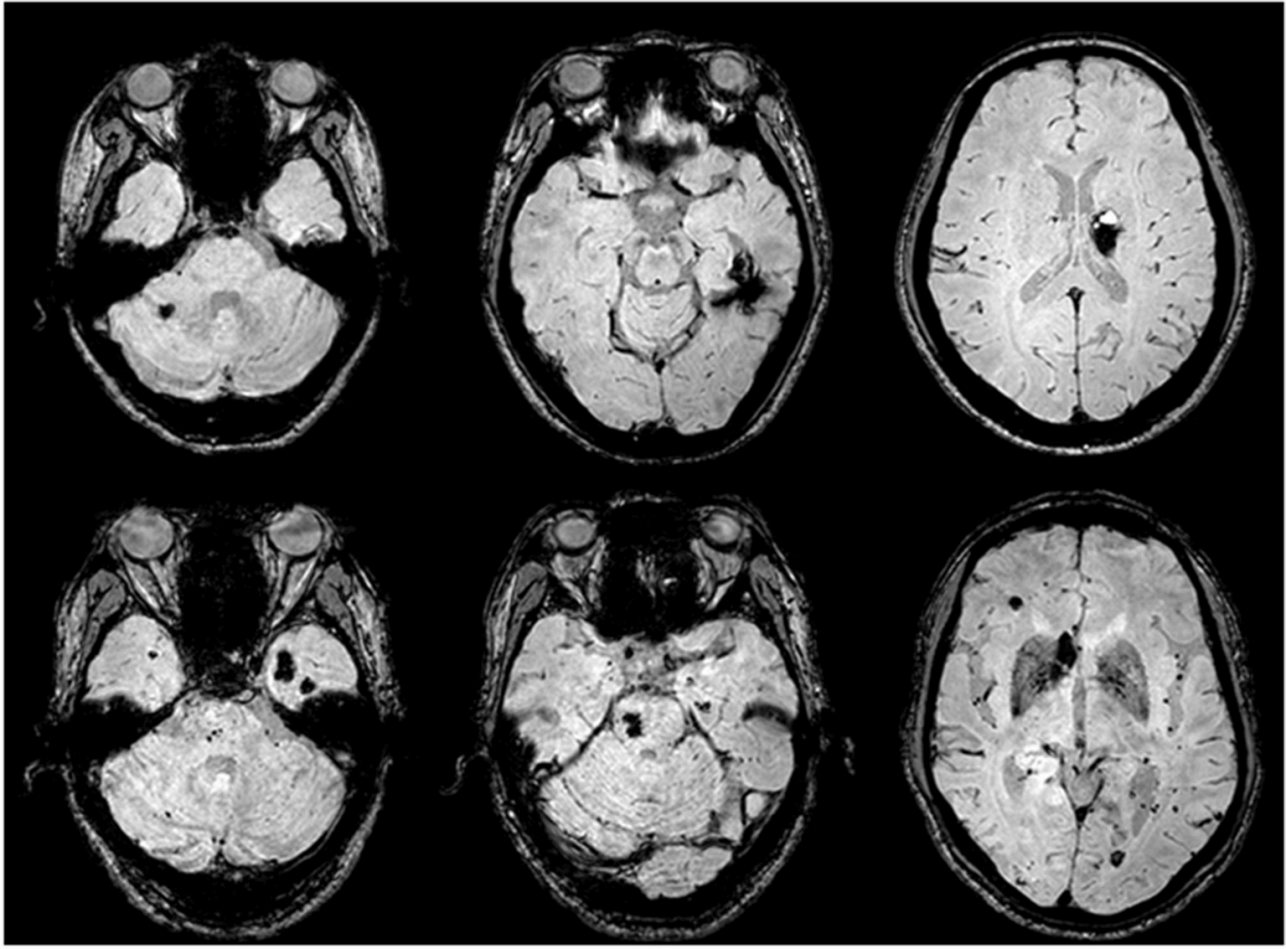
CCM lesions detected by SWI-MRI. The proband's SWIs (II:1) revealed dot and patchy low intensity lesions on temporal lobe, cerebellum, and thalamus (upper row). SWIs of the proband's father (I:1) demonstrated more widely-distributed multiple CCMs throughout the cerebrum, cerebellum, and pons (lower row).

The proband II:1 (Figure 3A) was a 34-year-old woman, presented with paroxysmal brief staring and loss of contact proceeded by uncontrollable weird thoughts for 2 years. Her minor seizures rarely evolved to generalize tonic-clonic seizures. She was administered multiple anti-seizure medications and several combinations, but the seizure frequency remained over 5 times per month. No positive neurologic sign was found. Whole brain MRI on March 6, 2014 showed three ultra-low intensity blooming lesions in the right cerebellar hemisphere and left basal ganglion on SWI, suggesting CCMs, and the “caput medusae”-like lesion on the left temporal-occipital cortex is compatible with venous malformation (Figure 4, upper row). VEEG showed abundant interictal spikes and slow waves over the left temporal region with phase reversals at T1 (data not shown). The patient received gamma knife radiosurgery targeting the left temporal lesion; however, the symptoms were not obviously improved. She was administered a combination of carbamazepine and topiramate. Comprehensive blood examinations including liver function, renal function, blood lipid, blood sugar, and blood electrolyte were normal.

The proband's father I:1 was 60-years-old and began to develop episodic fears followed by confusion and motionlessness about 20 years ago; these episodes lasted 2 to 4 min each time and occurred 3 to 5 times per year, but it progressed to 5 to 10 times per month during the past 5 years. Although he took multiple medications, the compliance was poor. Sudden falls accompanied by extreme convulsion and tongue-bites occurred occasionally. He was a heavy alcohol consumer. Neurological examination and routine blood examinations were normal. SWI sequence of brain MRI revealed eight visible CCMs on the left temporal lobe, bilateral frontal lobe, pons, and cerebellum (Figure 4, lower row). VEEG revealed frequent independent spikes originating from the bilateral temporal lobe with left side predominance (data not shown).

### Genetic Analysis

**Figure 5:**
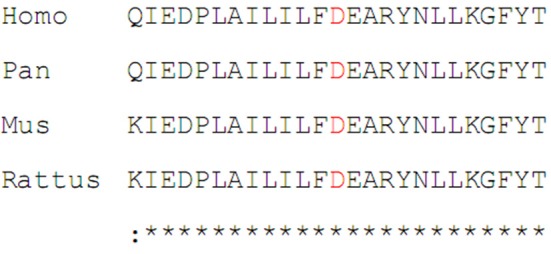
Alignment of the sequences of CCM1 protein in different mammalians.

In Family One, a novel *CCM1* genetic variant, c.1599_1601TGAdel, was found (Figures 1B,C). This genetic variant was present in family members II:1 and II:2 and is predicted to cause a p.Asp533del. Alignment of CCM1 protein sequences showed that the p.Asp533 is quite conserved in different mammalians, suggesting that the amino acid is important for CCM1 (Figure 5). No DNA samples were obtained from family members I:1 and I:2. In Family Two, a novel *CCM2* genetic variant, c.773delA (p.K258fsX34), was identified (Figures 3B,C) in family members I:1 and II:1, but not in family members I:2 and II:2. This genetic variant is predicted to cause a premature stop codon (TAG) at nucleotide 873 to generate a non-sense-mediated decay. The two genetic variants were not present in 100 normal controls, suggesting that they are causative genes for the clinical phenotype. These two genetic variants were not collected by the Human Gene Mutation Database (HGMD), ClinVar, and Gnomad database.

## Discussion

Familial CCMs exhibit more aggressive manifestations and an earlier age of onset when compared with sporadic cases, but familial CCMs are rare, accounting for ~6% of all CCMs. The disease is commonly presented with seizure, headache, and cerebral hemorrhage, but 11–44% patients might be asymptomatic for a long time, sometimes even lifelong (1). Depending on the symptoms and the findings in the proband, we justified that the proband's father or mother in Family One had CCM despite the absence of imaging evidence. CCM can be readily detected by MRI, particularly, the SWI with the characteristics of multiple dot or patchy lesions with markedly signal loss suggesting hemosiderin deposition. Other forms of vascular malformations, such as developmental venous anomalies, are thought to be closely associated with CCM and might co-exist with CCM as seen in the proband in Family Two.

Familial CCMs follow the autosomal dominant inheritance, and genetic variants of *CCM1, CCM2*, and *CCM3* genes are found in 53–65%, 15–19%, and 10–22% of the CCM families (26), respectively. In this study, we investigated two pedigrees with CCMs and identified one novel *CCM1* genetic variant (c.1599_1601TGAdel) and one novel *CCM2* gene genetic variant (c.773delA). These genetic variants cosegregated with the clinical phenotypes and were not present in the HGMD, ClinVar, and Gnomad database, nor in the 100 normal controls. Therefore, we can propose that these two novel genetic variants may account for the etiology. Our study expands the genetic variant spectrum of the patients with CCMs.

Currently, over 200 distinct germline genetic variants have been identified as causative genetic variants in the *CCM1, CCM2*, and *CCM3* genes (6). Most of the genetic variants are premature termination codons or large deletions (27, 28). Pathogenic deep intronic genetic variants of *CCM1-3* remain a controversial topic (29). Current evidence suggests that three CCM-gene-encoding proteins interact with each other to form a complex, the CCM signaling complex (CSC), which further interacts with other proteins (1). CSC can regulate various cellular signaling, including the signaling involved in cell-cell junctions (30). CCM proteins can also interact with other proteins through FERM domain, and subsequently be involved in various signaling pathways such as integrin signaling, Notch signaling, VEGF signaling, etc (31, 32). In addition to loss of function in CCM genes, non-genetic factors are also believed to play an important role in the pathogenesis of CCMs (32).

In Family One, the *CCM1* genetic variant, c.1599_1601TGAdel, is predicted to cause p.533Asp deletion. The proband and her sister had similar symptoms and radiological changes. Unfortunately, we did not get much information nor DNA samples from the proband's parents since they died early. Until now, more than 90 different *CCM1* genetic variants have been reported, with most of them causing premature stop codons; this suggests a loss-of-function mechanism (2, 33), which is responsible for CCM pathogenesis (31). The p.533Aspdel genetic variant is located in the FERM domain of CCM1 protein at the C-terminus (residues 420–736). Thus, the deletion might affect the binding of CCM1 protein to GTPase Rap1 through the FERM domain, and consequently interfere with the signal pathways involving in cell-cell junctions (30).

In Family Two, the *CCM2* genetic variant, c.773delA (p.K258fsX34), is predicted to cause non-sense-mediated decay. The patients showed typical CCM phenotypes, while different clinical manifestations were observed between the proband II:1 and her father I:1; the father I:1 had more severe clinical manifestations and CCMs lesions. This suggests that other unidentified *CCM* genes; for example, the putative *CCM4* gene; epigenetic factors; or other unknown variants of non-*CCM* genes may contribute to these differences in clinical penetrance between these two familial members. Thus, further studies in gene knockout animals are required to explore genotype-phenotype correlations of these *CCM* genetic variants and phenotype variability.

In conclusion, two novel genetic variants in *CCM1* and *CCM2* gene were identified in two Chinese families of CCMs, suggesting a causation of *CCM* genetic variant in CCMs. Our data enriched the genetic variant spectrum of *CCM* genes, which is useful for the early diagnosis of CCMs cases.

## Data Availability Statement

The raw data supporting the conclusions of this manuscript will be made available by the authors, without undue reservation, to any qualified researcher.

## Ethics Statement

Written informed consent to publish the report was obtained from each member of the family.

## Author Contributions

KW, DW, BZ, and GZ have actively participated in the data acquisition and interpretation, and they all commented and approved the final version of the manuscript. KW analyzed the data and drafted the manuscript. GZ designed the study and revised the manuscript.

### Conflict of Interest Statement

The authors declare that the research was conducted in the absence of any commercial or financial relationships that could be construed as a potential conflict of interest.
